# The Austronesian and the Micronesian Comparative Dictionaries as CLDF datasets

**DOI:** 10.1038/s41597-025-05301-4

**Published:** 2025-06-17

**Authors:** Alexander D. Smith, Robert Forkel, Lev Blumenfeld

**Affiliations:** 1 Fudan University, Institute of Modern Languages and Linguistics, Shanghai, China; 2 Max Planck Institute for Evolutionary Anthropology, Department of Linguistic and Cultural Evolution, Leipzig, 04103 Germany; 3 Carleton University, School of Linguistics and Language Studies, Ottawa, Canada

**Keywords:** Communication, Research management

## Abstract

The Austronesian Comparative Dictionary has served as an important resource for the comparative study of Austronesian languages since Robert Blust started its compilation in 1990. Likewise, the Micronesian Comparative Dictionary – an online database of Proto-Micronesian Reconstructions previously published in Oceanic Linguistics by Byron Bender and colleagues – is an important reference point for comparative Linguistics. The legacy, online versions of both dictionaries share an uncertain future, and both have not been available in a structured format, amenable to quantitative methods. Thus, to preserve the content of both dictionaries for the scientific record and to increase interoperability of the data, we undertook a conversion of the dictionaries to CLDF datasets. While programmatic access to the data within each dictionary already provides a new level of usability, the true potential of data in CLDF lies in interoperability *across* datasets. This is particularly useful for the two dictionaries presented here, because Micronesian languages belong to the Austronesian family and so the Micronesian data could potentially complement the Austronesian Comparative Dictionary. With the CLDF datasets we lay the groundwork for tackling this challenge.

## Background & Summary

The Austronesian Comparative Dictionary (henceforth “ACD”) is the largest data resource ever compiled for the comparative study of Austronesian languages, a group of 1,274 languages in the Asia-Pacific region that form one of the largest language families in the world^1,2^. It is part of the legacy of the late Robert Blust, who is responsible for the curation of nearly the entire existing ACD dataset. As a data source, the ACD contains linguistic data in the form of lexical entries from Austronesian languages organized into cognate sets with each set associated with one or more lexical reconstructions. The separate Micronesian Comparative Dictionary (henceforth “MCD”) is a similar data resource, originally compiled by Byron Bender, Ward H. Goodenough, Frederick H. Jackson, Jeffrey C. Marck, Kenneth L. Rehg, Ho min Sohn, Stephen Trussel, and Judith W. Wang, with a focus on the 21 Micronesian languages which form the Micronesian subgroup within the Austronesian family^3,4^. Both represent important data resources for research on Austronesian languages. For example, the most recent update to the ACD brought the total number of lexical entries to 119,768 individual words from 1,022 Austronesian language varieties organized into 8,161 cognate sets and associated reconstructions plus 2,364 non-lexical sets that include 302 sub-lexical cognate sets (so-called “roots”), and 2,062 other non-cognate groups (discussed further in our Methods section)^5^. While comparative dictionaries are often used to support arguments about proto-forms by referencing individual cognate sets^6,7^, the sheer size of the data in the ACD has also resulted in computational reuse, e.g. investigating evolutionary pressures on word formation^8^, or constructing networks of semantic evolution^9^.

The ACD and MCD previously existed online on sites hosted on the private servers of Stephen Trussel, the previous managing editor of both sources (https://trussel2.com/ACD and https://trussel2.com/MCD). After Trussel’s unexpected passing in 2020, however, the future of these datasets suddenly came into question. We began the process of transforming the ACD into a CLDF dataset in June 2021, after receiving a request from Blust. At the time, the ACD dataset was hidden in the data format of a customized closed-source curation application programmed by Trussel. With no access to the data on the hard disk of Trussel’s computer, our only option to access the data was by “scraping” the data from the HTML pages created as output of the curation application. In some sense, using these HTML pages as an authoritative source was also “more correct” because whatever use has been made of the ACD by the scientific community was based on the content of these pages rather than on the data as it lived in the curation application. A second consequence of our lack of access to the Trussel site is that the ACD now exists in two forms, the original Trussel site which now exists as a “heritage resource” and the new ACD online at https://acd.clld.org which will continue to receive updates.

To improve the re-usability of this dataset – and in particular support ongoing maintenance and curation of the data – we began building a completely new dataset with the ACD HTML as the principal data source. The new data format, CLDF^10^, is well suited for version control and provides a normalized data model for cognate-coded word-lists. CLDF can be used as storage format for the ACD, allowing tool-supported data curation as well as visualization and analysis using tools like the clld package. The transition to CLDF began in June 2021, but while the transition was still underway, Blust unexpectedly passed away in January of 2022. Thankfully, Blust was also taking care of arranging for a transition of editorial supervision and therefore began transitioning editorial control to Alexander D. Smith. This is where the current team, with Smith taking on the role of Blust and Forkel taking on the role of Trussel, took total control over the ACD database.

The MCD shares the fate of ACD in so far as it was published on Trussel’s platform and was incomplete, although, partly, in a more tractable way: originally published as “Micronesian Reconstructions” in two articles in Oceanic Linguistics, only the first was turned into the online database MCD. Since all languages spoken in Micronesia belong to the Austronesian family, theoretically, MCD would be a subset of ACD. Of course, considering the inevitable incompleteness of both datasets, this is not the case. Thus, with the work presented in this paper, we bring together both parts of the “Micronesian Reconstructions” in one dataset for the first time, and provide two major data collections for the study of Austronesian languages in a way that allows semi-automatic assessment of overlap and conflicts between the two sets, and lay the foundation for semi-automatic merging.

## Methods

### CLDF

We chose CLDF^10^ as distribution format for the datasets. CLDF is a package format for tabular data which organizes the data into mulitple tables following the principles of “tidy data”^11,12^. Thus, “each type of observational unit forms a table”. CLDF assigns a standardized meaning to several tables, for example the table listing the languages a dataset provides information about. Such tables are called *components* in CLDF and will be referred to with their component name – e.g. *LanguageTable* – in the following. In addition, CLDF allows the inclusion of non-standard types in “custom” tables. Such tables will be referred to using their file names in the following, e.g. cf.csv. CLDF is well-suited here, because its *Wordlist* module, together with the *CognateTable* and *CognatesetTable* components, support a data model that closely fits the data at hand. In addition, the possibility to encode references to objects in the dataset within text via *CLDF Markdown* (https://github.com/cldf/cldf/blob/master/extensions/markdown.md), allows us to also convert the sometimes extensive notes and comments provided for reconstructions in both datasets from somewhat idiosyncratic, hard-to-read HTML to basically plain text, without losing the links.

The ACD has been available as CLDF dataset since March 2023, and releases 1.1 and 1.2 of the data have been created from the CLDF as input data. Thus, we managed to gain experience with both the data model and curation procedures suitable for this type of data. Based on this experience and the work on the MCD as “experiment” to validate our assumptions, we streamlined the data model and present ACD 2.0 in this paper using a model that can be largely shared with MCD. In particular, ACD’s macro cognate sets – often subsuming subsets for multiple derived forms – have been split, with each subset now being represented by a row in *CognatesetTable*. The super-structure – which is typically missing in simpler comparative dictionaries – is preserved by adding a custom table etyma.csv to the dataset and a foreign key from *CognatesetTable* pointing to it. We also created a uniform representation for groups of forms which are not related by cognacy, and thus, do not give rise to a reconstruction. In ACD, such sets are the ones in the “noise”, “near”, “loan” and “root” categories. In MCD, groups of forms that are of interest in relation to a cognate set – for example forms that are considered “candidates” for membership, but some kind of irregularity is grounds for exclusion – are listed after each cognate set, but not categorized any further. Since these groups may carry additional annotation, e.g. a comment, we list groups in a custom table cf.csv and members of groups in a custom table cfitems.csv, as the association table between *FormTable* and “cf” table.

In order to make sure the data model is applied consistently in both datasets, we created a Python package pyetymdict^13^ with functionality to create conformant CLDF data. This package serves the additional purpose of documenting the shared semantics of the custom types used in the datasets, thereby putting etymological dictionaries on track for further standardization within CLDF (see https://github.com/cldf/cldf/issues/163).

### Parsing the HTML pages of ACD

**Fig. 1:**
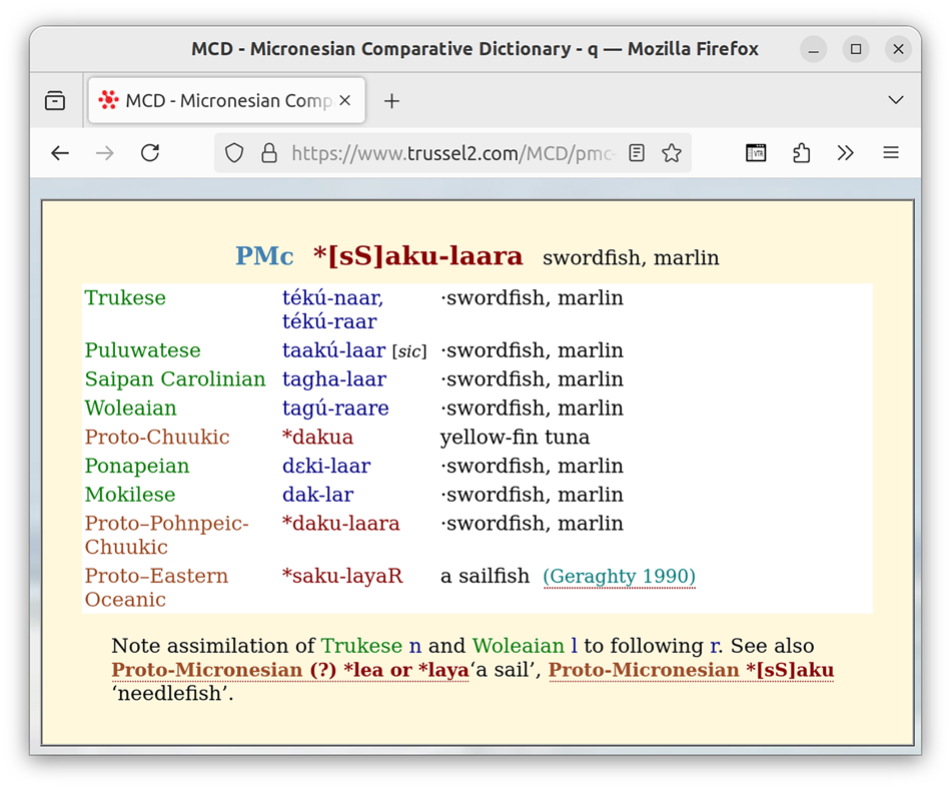
A reconstruction from MCD as displayed in the legacy online database. While the MCD has a simpler structure than the ACD – lacking collections of reconstructions – the basic structure of individual reconstructions (or cognate sets) is the same: A tripartite header, giving the reconstruction level, the proto-form and the reconstructed meaning, is followed by a table of reflexes and often a note.

**Fig. 2:**
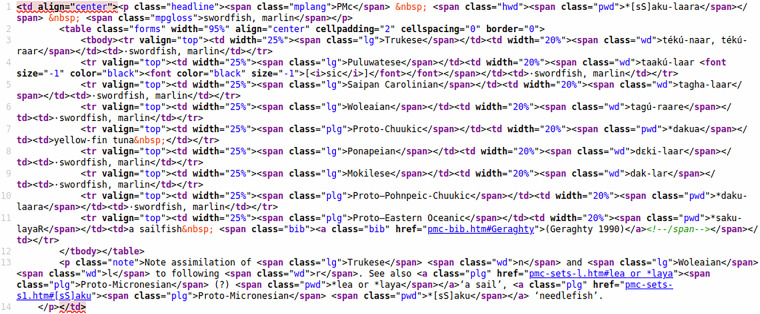
This figure shows the HTML source of the MCD reconstruction from Fig. 1. While some of the structure of the data is well reflected in the HTML – e.g. the table of reflexes is encoded as an HTML table element – some information is somewhat implicit, e.g. the “[sic]”, qualifying the word form “takú-laar” is simply nested within the td element for the word form.

While ACD 2.0 has been created from the CLDF data of ACD 1.1 as input, the bulk of the data is still ultimately the result of parsing the HTML pages of the legacy site (see Fig. 1 for an example from MCD). Thus, we describe this parsing process as the basic method to obtain the data. Details about Trussel’s workflow for creating the HTML version of ACD are unknown, but it seems clear that the majority of the HTML has been somehow created by the curation application. Fortunately, this process created some sort of semantic markup, i.e., some level of data structure could readily be inferred from corresponding HTML markup (see Fig. 2). Unfortunately, the HTML also looked as if different parts were created using different versions of Trussel’s curation application. For example, word forms are variously marked up using tags of the form <wd> or <span class="wd">. Unknown tags are allowed per the HTML specification^14^, and can generally be recognized and processed using off-the-shelf HTML parsers like Python’s htmllib. So this variation just required more flexible handling of the parsed HTML structure. It also turned out, though, that some of the HTML was not well-formed. For example word forms containing reserved characters in HTML like < were not escaped properly. Thus, the parser we implemented had to apply fixes at various stages of the parsing: To turn the input into well-formed HTML before using a standard HTML parser and to make sense of non-HTML tags when post-processing the output of this parser.

Another set of inconsistencies stems from data objects not being rendered as HTML in the same way across different types of pages. For example the required escaping of reserved characters was applied to word forms on the cognate set pages – but not in the lists of words on the language pages. Finally, it seems that data updates not always resulted in recreating **all** parts of all HTML pages – leading to inconsistencies, for example between the cognate set statistics given at the top of the cognate set pages and the actual item counts.

Since parsing the ACD’s HTML pages was a one-off effort, we made our code openly accessible at https://github.com/lexibank/acd/tree/v1.0/acdparser, but did not put it into a proper Python package. While we did derive the code for parsing the MCD pages (see below) from the one written for the ACD, the various adaptations we had to introduce again corroborated that there is no generic value in this code, meriting packaging and distribution.

### Identifying languages

The original ACD had no official method for identifying languages and linking them to a standardized nomenclature for language organization such as ISO-639-3 or Glottolog^2,15^. We therefore had to standardize the database by associating the named languages of the ACD with both ISO and Glottolog language codes.

We paired language names with matching Glottolog entries with simple automatic matching. Glottolog already links Glottocodes^16^ with ISO codes, so we were able to associate languages with both Glottolog and ISO simultaneously. The output of the automatic matching was later double checked by Smith with manual corrections made wherever necessary. The standardized language codes are available in the *LanguageTable* of the CLDF dataset. All but two languages ("Malagasy (Provincial)” and “Malay (Borneo)”) could be matched to a Glottolog language, dialect or subgroup in this way.

It should be noted that we expect this language identification to pay off in multiple ways. In particular, since other big resources of language data for Austronesian languages like the ABVD^17^ are available in CLDF as well, we hope to either inform the language identification in these resources on the basis of the ACD or to be able to properly investigate overlap between the datasets.

### Parsing the HTML pages of MCD part 1

**Fig. 3:**
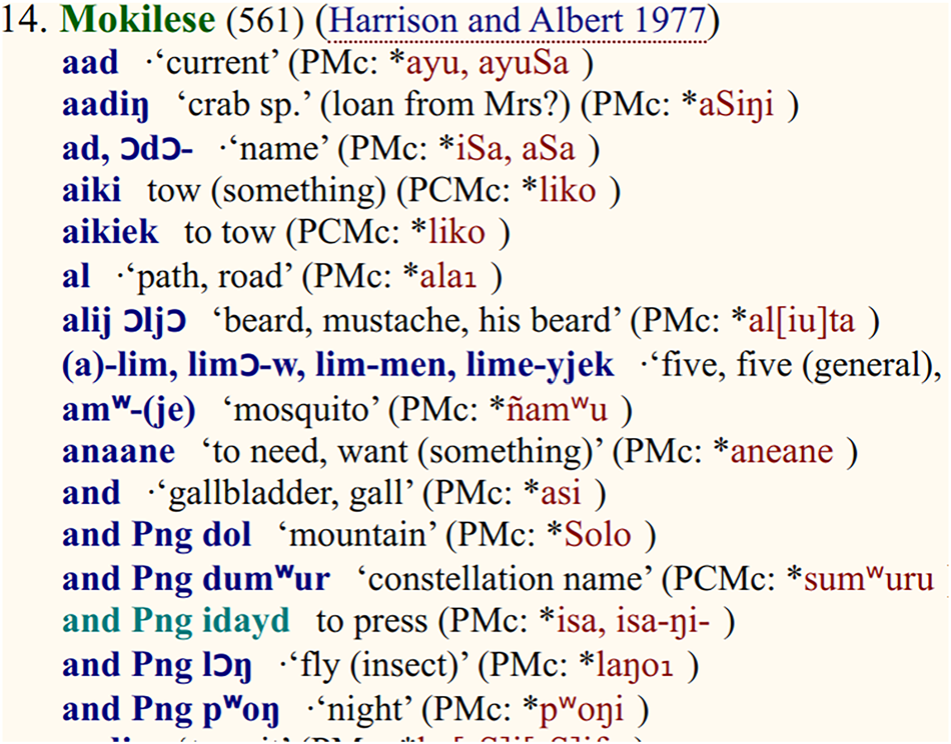
Five Pingilapese forms are listed together with identical forms in the Mokilese wordlist.

**Fig. 4:**
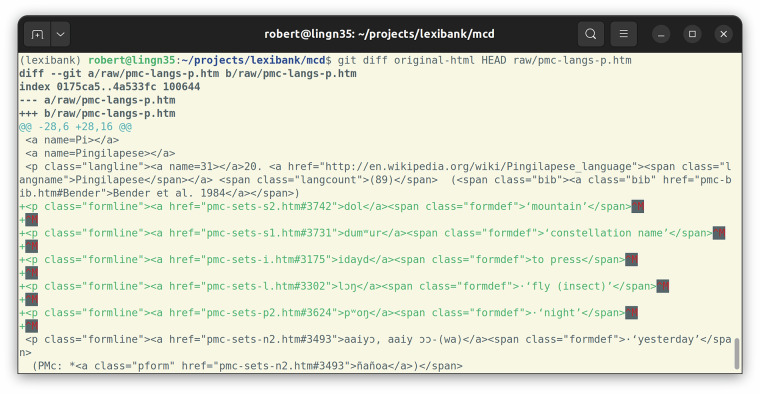
Using the git diff command, we can see how the HTML page containing the Pingilapese wordlist has changed by inserting the five words that were mixed into the Mokilese wordlist.

Thanks to the “Proto-Micronesian Reconstructions – 1”^3^ being available online in a format similar to the one of the ACD, much of the code used to parse the ACD pages could be reused to parse the MCD. Also, thanks to our experience with the ACD, we checked the correctness of the parsing by comparing our results with summary statistics listed in the HTML pages (see below). Some of the inconsistencies discovered by this check (see Fig. 3) were resolved by editing ("fixing”) the HTML pages in order to keep parsing simple. Since we keep these HTML pages in the dataset’s git repository as well, changes to these pages can transparently be tracked (see Fig. 4).

### Parsing the data of MCD part 2

While “Proto-Micronesian Reconstructions – 2”^4^ was published not long after the first part, it was never included in the online MCD. Thus, converting the reconstructions to a structured format started from a plain text file extracted from a PDF version of the article in Oceanic Linguistics. Fortunately, the text format was consistent enough to extract data in a semi-automated process. Nevertheless, several corrections were necessary: First, due to no information about the fonts used in the PDF being available, the Unicode text extracted from the file needed to be corrected, for example fixing incorrectly interpreted glyphs for ligatures or other non-ASCII characters.

Then, the paragraphs containing reconstructions could be detected as the ones starting with the abbreviation of the proto-language and a reconstructed form indicated by a leading asterisk. Excluding paragraphs that only referenced other reconstructions (such as “PCk *pwaya ‘swarm of fish’: See PCk *pweya or *pwaya ‘swarm of fish’.”), we arrived at 716 reconstructions. These reconstructions were put into a text file and then (semi-automatically) turned into tab-separated chunks. I.e., we created a table of the form Table 1

**Table 1:** Reconstructions from MCD part 2 were converted semi-automatically into the tabular format shown in this table.

language	form	gloss	cognates	“cf” forms	“see also” forms	notes
PPC	*diwa-ike	‘ninety’	Chk ttiwe ‘ninety’ …		See PMc *Siwa ‘nine’.	

This format served as the input for fully automated data extraction.

Both the HTML and the tabular data are read and parsed when the CLDF-creation pipeline is run (see below).

### Orthography Profiles for MCD

To maximise interoperability – both with the ACD and other lexical data – we standardized the transcriptions of word forms in the MCD using *orthography profiles* (see chapter 7 in^18^). Since the number of languages in the MCD is manageable in comparison to the ACD, we created per-language orthography profiles, which increases transparency because each profile represents the full grapheme inventory for the transcriptions of words in one language (see for example the profile for Arosi at https://github.com/lexibank/mcd/blob/main/etc/orthography/arosi.tsv).

### The processing pipeline

The processing pipeline to create CLDF datasets from the “raw” (pre-processed) data is implemented in both cases using the cldfbench software^19^. In addition to making it easy to integrate data from reference catalogs such as Glottolog, cldfbench supports a repository layout which transparently mirrors provenance, with data from the source publications in a raw directory and additional processing information such as orthography profiles in a etc directory.

The different “life cycles” for the two datasets lead to differences in the maintenance of the processing pipeline. The MCD reflects the content of two published works which will not receive any updates by the original authors. Thus, CLDF creation can always start from the “raw” data, possibly extended with routines to fix errata. The ACD on the other hand will receive updates of its content beyond merely fixing errors. So CLDF creation for each new release will start from the data of the previous release, integrating updates in the process. While MCD still requires code to parse the HTML data, this is no longer the case for ACD. Since the code implementing the CLDF creation is versioned together with the data, recreating previous releases is still possible.

## Data Records

Both datasets are archived with and available from the Zenodo repository^5,20^.

At the highest level, the two datasets represent language genealogies, i.e., hypotheses about composition of and genealogical relations within a language family. This hypothesis is implied by etyma (also called “cognate sets” in ACD or “reconstructions” in MCD), groups of word forms from languages of the proposed family that share the same etymology and can thus be used to reconstruct a proto-form belonging to an ancestral node in the family tree via the comparative method^21^.

**Fig. 5:**
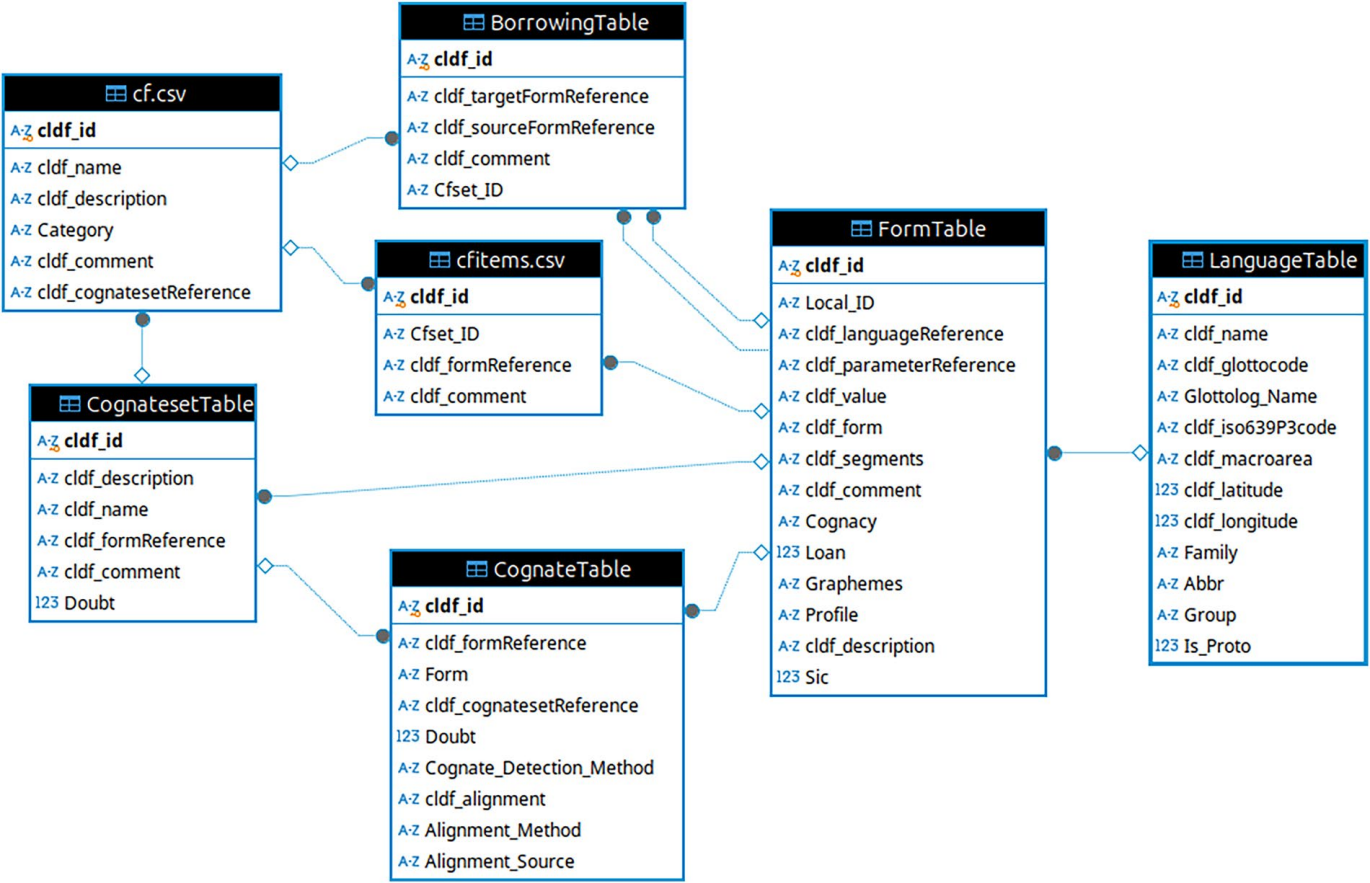
This figure shows an entity-relationship diagram of the main tables of the CLDF data model. The two tables on the left describe sets of forms. The three tables in the middle group forms into three types of sets, “borrowings” (or “loans”), “cf” (or “see also”) and “cognates”. Forms – the rows in *FormTable* hold the lexical data and the relation to (proto-)languages. The direct relationship between *CognatesetTable* and *FormTable* links cognate sets to a reconstructed proto-form. Some tables have been omitted for simplicity, e.g. the *ParameterTable*.

**Fig. 6:**
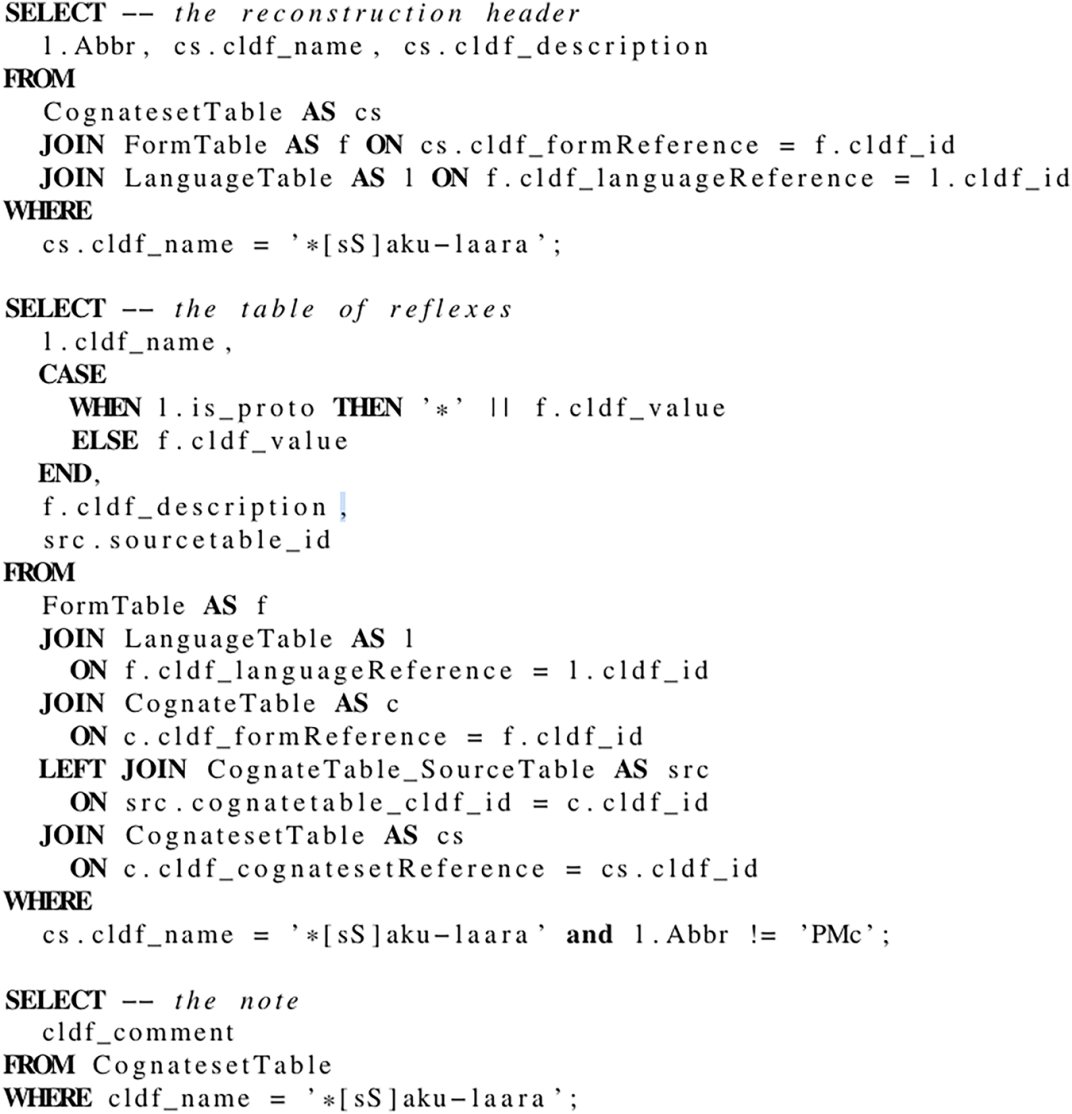
This figure shows three SQL code snippets to extract the data shown in Fig. 9 from the multi-table CLDF dataset with relations as shown in Fig. 5.

With the help of the CLDF data model, these rather complex etyma can be broken down into suitably linked, reusable building blocks. Thus, we represent the two datasets as CLDF Wordlists with CognateTables (see the entity-relationship diagram of the data model in Fig. 5 and an example for re-assembling a reconstruction from this multi-table data in Fig. 6).

While denormalization is often useful to improve “readability” of a complex dataset (we could, for example, add a flag to each form signaling whether it is a proto-form – multiplicating the information from the language table), the redundancy it creates can get in the way of manageability. Thus, we limit redundant information to two cases: 1) Reconstructed proto-forms serve also as labels of the corresponding cognate sets and 2) the meaning descriptions of forms are stored in the *FormTable* (as column cldf_description) as well as in the parameter table (omitted in Fig. 5). The latter decision was taken to allow for later standardization of meanings while keeping the – often language-specific – meaning descriptions from the source.

The objects of investigation of the datasets are the languages which are compared, represented as rows in a LanguageTable with links to the bibliography listing sources for the lexical material, serialized as BibTeX file. Since proto-forms are part of the datasets, too, we include the respective proto-languages in the *LanguageTable* (flagged as such).

The “smallest” unit of information in the CLDF datasets are individual meaning descriptions, represented as rows in the ParameterTable. It should be noted that due to the immense variation of meaning descriptions in the sources no attempt has been made (yet) to normalize these. Thus, the “concept lists” of both datasets have more than half the number of entries as there are individual forms in the datasets. Cursory investigation of the meaning descriptions promises a possible reduction by an order of magnitude, though, and re-use of this kind of data in projects like EvoSem^9^ might provide enough incentive to tackle this issue.

The lexical material – word forms extracted from sources as well as reconstructed proto-forms – is represented as rows in the FormTable. Forms are provided as given in the legacy datasets as well as in a somewhat standardized transcription obtained by using orthography profiles as explained above. Rows in *FormTable* are linked to a language and a meaning description transparently via *languageReference* and *parameterReference* columns.

A full description of all tables and columns in the datasets is available as part of the dataset package at cldf/cldf-metadata.json. Human-readable versions of this metadata can be created automatically and are available at cldf/README.md and in the repositories at https://github.com/lexibank/acd/blob/main/cldf/README.md for the ACD data and https://github.com/lexibank/mcd/blob/main/cldf/README.md for the MCD data.

### Identifiers

Complex data of the type presented here – with relations between multiple tables – critically depends on identifiers (or “keys”) for all objects^12^. With data curated largely “by hand”, *natural keys* are often preferred, i.e., keys derived from a unique property of the identified object. Here, cognate sets are identified in the legacy HTML pages using the reconstructed proto-form as key – if necessary, suffixed with a disambiguation marker. The downside of natural keys is that they must be changed (or lose their mnemonic advantage) if the underlying property changes (e.g. because a reconstruction is corrected). Thus, Trussel’s system also assigned numeric *surrogate keys* to reconstructions (and languages). We kept these numeric keys as identifiers of the respective objects in the CLDF dataset, making it possible to recreate object-specific, “deep” links into the legacy sites.

### Etyma

While the data model outlined above matches the model used to represent many cognate-coded lexical datasets^22^, the semantics of the cognates differs slightly: In the datasets presented here, cognacy is not interpreted narrowly, requiring exactly matching meaning. Although, generally, words grouped in a cognate set will have similar meanings. Thus, rows in *CognateTable* group forms into sets based on shared etymology. (This “semantic looseness” enables re-use of these datasets to investigate evolution of semantics.) Additional data related to such a set is provided in a *CognatesetTable*, e.g. notes and links to references. Crucially, though, cognate sets link forms to a reconstruction which is justified by the forms. This reconstruction is represented as a row in *FormTable*, linked to a proto-language. The reconstruction is linked to the *CognatesetTable* using a *formReference* property.

In particular in the ACD, etyma are also often accompanied by rich notes, linking to other objects in the dataset like lexical items, reconstructions or languages. We preserve these notes – including the links – by converting them to *CLDF Markdown* (see https://github.com/cldf/cldf/blob/master/extensions/markdown.md), a variant of the text-markup language Markdown with links to objects in a CLDF dataset embedded in Markdown links. In the ACD, most of these notes are attached to the super-sets, and therefore available as *Comment* column in the table etyma.csv. In the MCD (and for sub-set level annotations in ACD) they are stored in the *Comment* column of *CognatesetTable*.

### Reconstruction tree

**Fig. 7:**
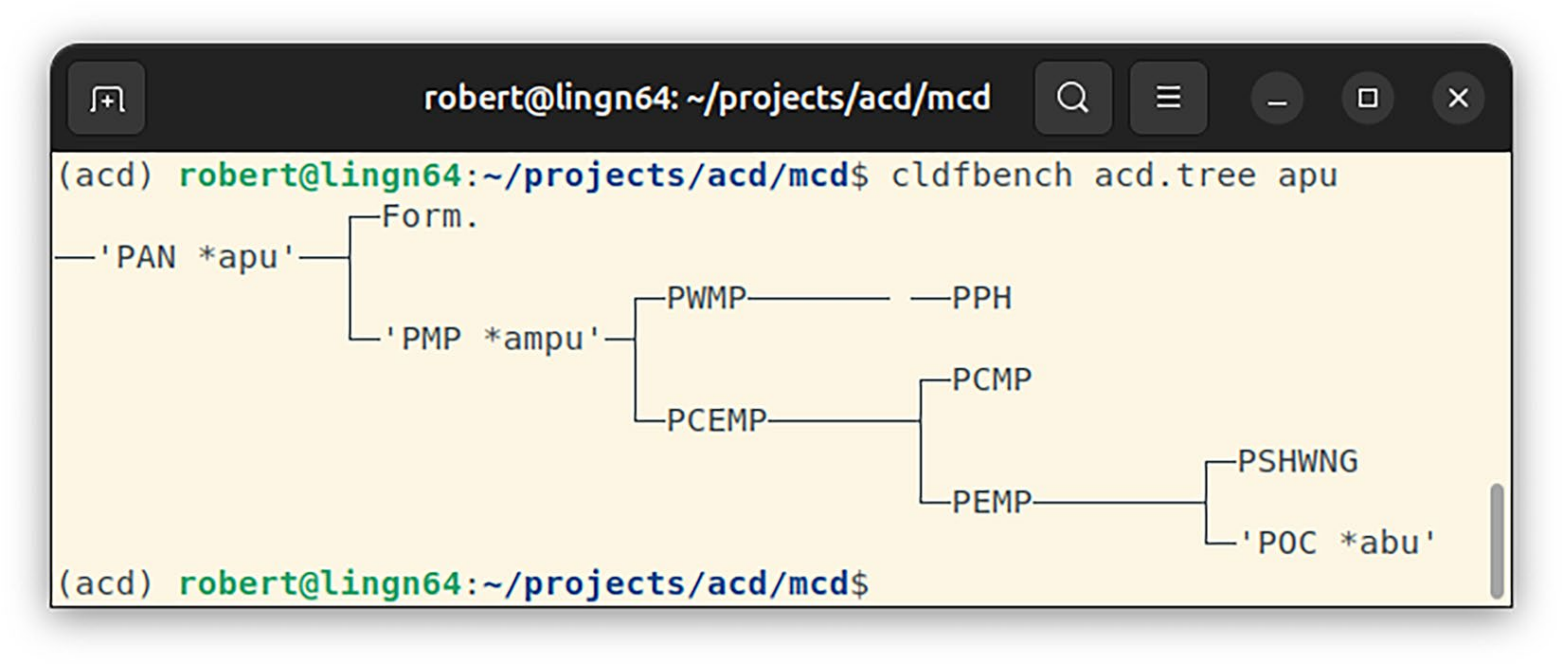
Screenshot of a terminal window where the acd.tree command is run to plot the reconstructions of Proto-Austronesian *apu on the Austronesian language group tree. Plotting reconstructed proto-forms on the assumed classification tree allows investigation of sound changes together with the direction of change provided by the tree.

Both datasets reconstruct proto-forms on multiple levels, i.e., at multiple nodes of a tree assumed to represent the (coarse) classification of the group of languages under investigation. This tree is important both as assumption underlying the reconstruction work and as device to interpret the results of the analysis (see Fig. 7). CLDF provides a way to include a language tree in a dataset, using a *TreeTable* component, linking to the actual tree in Newick format (see https://en.wikipedia.org/wiki/Newick_format) in a row of a *MediaTable*.

### Other groups of forms

Both the ACD and the MCD provide groupings of lexical items other than cognate sets. The main motivation to include such “by-products” of the core reconstruction work is to keep track and provide a log of that work. Or as Blust stated: “By including a module on “Noise,” I can show that I have considered and rejected some possibilities that might be entertained by others.”^1^

Such groups of forms are represented in the CLDF datasets as rows in a table cf.csv, and group members linked to these rows in the table cfitems.csv, or in the standard CLDF component *BorrowingTable* for the case of groups of loanwords. In the MCD each of these groups is explicitly listed under a reconstruction, and this relation if reflected by a foreign key in cf.csv referencing *CognatesetTable*. In ACD this is not the case, but groups are additionally categorized as “noise”, “near”, “root” or “loan”.

### Other annotation

The ACD contains various other annotations of its core data. Noted irregularity of forms in the context of a cognate set is annotated with flags signaling *metathesis* or *assimilation*. These flags are available as columns in the *CognateTable* since they describe a property of the form in relation to a cognate set.

The ACD also annotates “doublets” and “disjuncts” (see^1^, p.503), i.e., groups of variants of reconstructions. In the legacy HTML pages, this annotation was realized as cross-references between members of these groups. This made updating the annotations error-prone, because adding a new doublet to a group required adding cross-references to each member of the group. We consider both relations, doublet and disjunct, to be symmetric and transitive and annotate group membership of a reconstruction by storing a group identifier in the respective column of *CognateTable*. This allows adding members to a group without having to change the data of any of the other members.

Lastly, since the ACD explicitly tries to “supplant Dempwolff (1938) as the primary source of historical data on the entire Austronesian language family”^23^, it references corresponding etymologies in Dempwolff (1938)^24^ whenever the etymology was deemed to not fit the criteria for “canonical” reconstructions in ACD – and thus the group of related forms was relegated to a “noise”, “near” or “loan” set. Consequently, this annotation is available as *Dempwolff_Etymology* column in the table cf.csv.

Both, ACD and MCD employ the common – if somewhat unsatisfying from the perspective of automated data reuse – practice of marking uncertainty with question marks intermingled with the data. In cases such as “Kanakanabu nimu (?)” (see https://trussel2.com/ACD/acd-s_a1.htm#31575), where we could not decide whether uncertainty of the cognacy or uncertainty of the existence of the word form was implied, we just left the “(?)” annotation as part of the *Value* in *FormTable*. In cases of proto-forms like “Proto-Eastern Oceanic (?) *waqe” (see https://trussel2.com/MCD/pmc-sets-w.htm#wa), we interpreted the question mark as meaning uncertainty of the reconstruction, and thus converted it to a *true* value in the *Doubt* column of the corresponding row in *CognateTable*.

## Technical Validation

The validity and usefulness of the two datasets for research in Comparative and Historical Linguistics is established by their citation record. The main contribution of the derived datasets presented here consists in making the data from ACD and MCD available in a format that is suitable for re-use, including automated re-use – and in particular as a basis for ongoing data curation.

Thus, we have to make sure that 1) the content of the datasets corresponds to the content of the previous formats which were meant for “human consumption” only and 2) our datasets are in fact re-usable using a broad range of software tools, including software that can exploit the relational nature of the data.

### Completeness of the datasets

In order to assess the completeness of the datasets, we compare the results of our conversion procedure against published counts. For the ACD, the legacy online version contains two sets of such counts: 1) The cognate set pages list the numbers of reconstructions per first grapheme of the reconstruction and 2) the language pages list the number of words per language.

We already compute the initial grapheme of the main reconstructions (in a way compatible with the one used for the Trussel site) when creating the CLDF data, so computing the corresponding counts is simple. Adjusting these counts, accounting for the additions since ACD version 1.0, and comparing against the counts from the Trussel site, we only get minimal differences for six initials. For “d”, “S” and “R” we seem to have missed one reconstruction while for “h”, “m” and “r” we seem to have gained one. Considering our concerns regarding the consistency of data on the legacy pages (and the fact that we got the total count exactly right), we take this as ascertainment of the completeness of our data with regards to cognate sets.

**Fig. 8:**
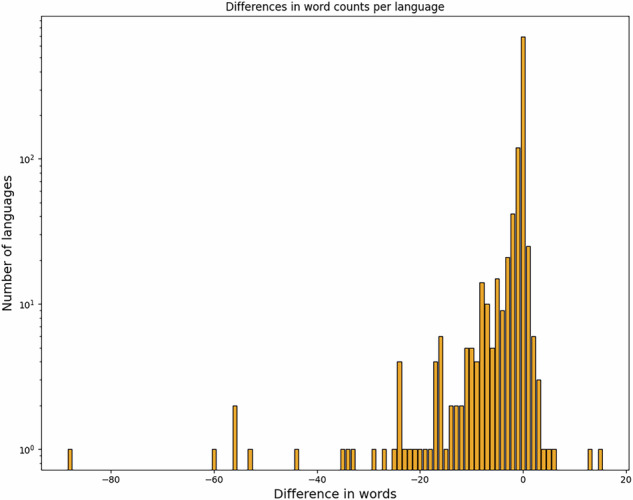
Agreement between numbers of forms per language as stated in the legacy HTML pages and as computed from our CLDF data. The y-axis is scaled logarithmically, so more than two thirds of the languages show no difference (although this might be due to splits and mergers canceling each other out). The most extreme outlier – Maranao, with 88 words less in our data was cross-checked manually, and indeed had the expected amount of duplicates.

Counting the number of words per language in our dataset is equally simple; exact matches with the counts on the legacy HTML pages cannot really be expected, though, because we 1) identified words with the same form and meaning description when parsing the data and 2) split items where multiple variant forms were given (e.g. as in “tenzeg, te(n)dek”) into multiple words. Still, the agreement is quite close as can be seen in Fig. 8.

In theory, all words in the ACD are members of at least one set. So, if the same is true for our dataset, this would establish fully accurate parsing of the source data. It turns out, though, that 93 words in the CLDF data are not linked to any set. But being able to easily compute these helped us identify minor problems with the HTML pages – and consequently with the parsing. Since the ACD is still maintained and updated, rather than complicating our parsing code we simply list these issues as errata, to be fixed with the next release (see https://github.com/lexibank/acd/milestone/2).

As with the ACD, the legacy online MCD lists all word forms twice, once on the “Languages” pages (https://www.trussel2.com/MCD/pmc-langs-a.htm) – in a list of all words per language – and once on the “Cognate Sets” pages (https://www.trussel2.com/MCD/pmc-sets-a.htm), listed as reflex of a reconstruction. This allows us to check the **correctness** of our parsing by making sure the two sets of forms parsed from two different sets of pages are identical.

There are also two “summary statistics” available to verify the **completeness** of our results. The total number of etyma is given as “some 980 reconstructions” in the introduction (https://www.trussel2.com/MCD/pmc-intro.htm). Thus, we should expect to parse 980 reconstructions from the cognate set pages. Considering that the introduction was taken from the print publication, and some data curation by Stephen Trussel has been going on between 2003 and 2013 – the latest year any of the HTML pages have changed – the 991 etyma we extracted from the HTML can be assumed to be the complete set. The number of words per language is given on the language pages. While these numbers matched our numbers of parsed words for many languages, we still ended up with different numbers for 20 languages. These differences are listed in the repository (https://github.com/lexibank/mcd/blob/main/lexibank_mcd.py) and are due to two issues: We merge duplicate forms, i.e. words with identical form and meaning. Such duplicates show up in MCD’s wordlists because these wordlists are rather to be read as “reference lists”, i.e. they list occurrences of words as support for reconstructions. Often, duplicates are due to the same word being listed in “Cf tables” of two etyma. But some are also listed as proper reflexes of different etyma, hinting at a potential issue with the data. It should be noted that within the CLDF data such cases are a lot easier to detect, allowing more principled handling in analysis. While duplicates account for all cases where we parsed **less** words than expected, there are also four languages where we parsed **more**. These cases are caused by forms from one language being listed **within** entries of another language as shown in Fig. 3. Since we wanted to fix these words without having to change our parsing pipeline, we edited the downloaded HTML pages (https://github.com/lexibank/mcd/tree/main/raw), moving the forms in question to the correct language. Since the downloaded HTML pages are kept in the dataset’s git repository, changes can be inspected using the git software (see Fig. 4).

### Correctness of the datasets

**Fig. 9:**
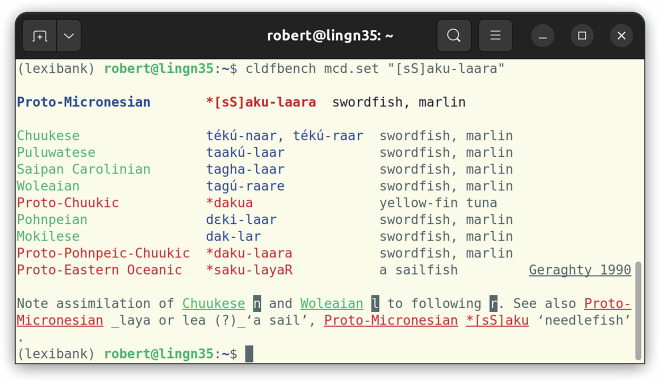
The reconstruction from MCD shown in Fig. 1 displayed on the UNIX shell, using a small Python program to read and assemble the data from the CLDF dataset. Note that all parts of the data structure can be recovered, also within the text of the note. Also note that language names have been normalized, specifying the proto-language of the reconstruction as “Proto-Micronesian” rather than “PMc” and using the standard name “Chuukese” instead of the alias “Trukese”. SQL code, to be run on the SQLite version of the MCD dataset, which is roughly equivalent to this Python program, is shown in Fig. 6.

Checking the correctness of the parsing was an essential and continuous task during the development of the parsing code. So we will limit the validation of correctness in this paper to exemplary samples. A list of such samples was already used in the Research Note^1^ to describe the contents and features of the database. In order to allow for comparing the examples in the Research Note with the data in our dataset easily, we implemented a cldfbench subcommand to display a cognate set in the UNIX shell following the typographic layout used by Blust. The output of this tool and the examples from the Research Note are listed on a page in the repository (see https://github.com/lexibank/acd/blob/main/VALIDATION.md, created by the code in https://github.com/lexibank/acd/blob/main/acdcommands/validation.py). Figures 1 and 9 show an example of using the tool to display the construction *[sS]aku-laara from MCD (https://www.trussel2.com/MCD/pmc-sets-s1.htm#[sS]aku-laara).

### Re-usability of the CLDF datasets

Re-usability of typed, relational data depends on correctness and consistency. I.e., declaring that a column of a table in the dataset holds values of a certain data type is only useful if it is guaranteed that the data conforms to this specification; and similarly, relations between tables are only useful if relational integrity of the dataset can be assumed. This type of basic technical validation is already provided through the CLDF package and ecosystem. Since CLDF also specifies domain specific data types such as Glottocodes as identifiers for languages, CLDF validation also provides basic semantic interoperability.

"Traditionally”, re-using the data in these datasets meant citing a reconstruction as reference for inferences drawn from it. While this use case was supported by the legacy databases, providing reconstruction-specific URLs, they also created the “moving target” problem: Since the databases were subject to – potentially – continuous updates, visiting a cited URL did not guarantee that the visitors saw the cited information. We hope to improve the accuracy of the scientific record in accordance with emerging best practices in the field^25^ by releasing fixed versions of the datasets, on properly citeable platforms like Zenodo, thereby making references durable.

### A baseline and foundation for the future

Finally, it should be noted that the work presented here is particularly useful because it lays the foundation for the future. As Blust wrote in 2013^1^,

projects like this enter the public domain, and […] the project itself need not be limited to a single person or a human lifetime.

With our dataset it becomes possible for the public to programmatically assess the coverage and consistency of the data and thus to help fill in the gaps and weed out the errors. Among the things that can be readily computed now – although fixing will need more work and editorial decisions – are

- duplicate forms and reconstructions,
- forms that are cited as reflexes of multiple reconstructions,
- forms that are cited as reflexes of “later” proto-forms.

While remarkably few, such inconsistencies corroborate our belief that resources at the size of the ACD can only be curated with an adequate data model and tools like CLDF which allow automating consistency checks.

## Usage Notes

One could argue that – at least with respect to the ACD – we merely recreated the status that was already achieved in Trussel’s database system: The ACD, available as properly structured data accessible for programmatic use. But this would ignore important benefits gained through the CLDF format. The machine readable representation of the datasets is no longer the internal memory of one application running on one machine, but a set of files following an open specification published in a research data repository.

Due to the highly relational nature of the data in this dataset, using the records as serialized in the CSV files of the CLDF package is not trivial. The CLDF metadata, on the other hand, provides information about the relations between the tables in the model, and can thus be used to inform processing. This is the route taken by the *orm* (for “object-relational mapping”) module of the pycldf package. It provides a Python *API* ("application programming interface”) to the data which allows “drilling down”, i.e., resolving relations into related objects (see https://github.com/lexibank/acd/blob/main/USAGE.md#using-pycldf). On computing platforms other than Python, similarly convenient access is ensured because each CLDF dataset can be converted to an SQLite database. Virtually every computing environment allows running SQL queries on SQLite databases, e.g. RSQLite in R or sqlite3 on the UNIX shell. (For instructions on converting the dataset to SQLite and examples of running queries, see https://github.com/lexibank/acd/blob/main/USAGE.md#using-sqlite.)

While the tools mentioned above may fall outside the toolbox of “traditional” linguists working with etymological dictionaries, it is worth noting that both tools and data formats presented here are fully in line with emerging best practices for research computing in general. In particular, following through the handful of lessons from the excellent *Software Carpentry* intiative (see https://software-carpentry.org/lessons/) will equip researchers to make full use of the data presented here.

## Data Availability

The Python packages used to create the CLDF datasets are listed in each dataset’s cldf/requirements.txt file. To provide uniformity of the data model for etymological dictionaries on top of CLDF Wordlists, we implemented the shared schema in the Python package pyetymdict^13^, which is available from the Python Package Index (https://pypi.org/project/pyetymdict/) and archived on Zenodo (10.5281/zenodo.14534564). The code implementing the dataset creation as well as the technical validation described above is available in each dataset’s GitHub repository (and thus archived together with the datasets on Zenodo).
